# A Pediatric Investigators Collaborative Network on Infections in Children (PICNIC) multi-centre Canadian descriptive analysis of *Haemophilus influenzae* bacteremia in children: Emerging serotypes

**DOI:** 10.14745/ccdr.v49i09a02

**Published:** 2023-09-01

**Authors:** Craig Frankel, Joan Robinson, Sarah Khan, Mohammad Alghounaim, Jane McDonald, Alison Lopez, Sergio Fanella, John Gunawan, Jacqueline Wong, Jeannette Comeau, Jennifer Bowes, Robert Slinger, Angela Kalia, Ashley Roberts, Kirk Leifso, Marina Ulanova, Michelle Barton

**Affiliations:** 1 Department of Paediatrics, Children’s Hospital, London Health Sciences Centre, London, ON; 2 Department of Pediatrics, Stollery Children’s Hospital, Edmonton, AB; 3 Department of Pediatrics, McMaster Children’s Hospital, Hamilton, ON; 4 Department of Pediatrics, Montreal Children’s Hospital, Montréal, QC; 5 Department of Pediatrics and Child Health, Winnipeg Children’s Hospital, Winnipeg, MB; 6 Department of Pediatrics, IWK Health Centre, Halifax, NS; 7 Department of Pediatrics, Children’s Hospital of Eastern Ontario, Ottawa, ON; 8 Department of Pediatrics, BC Children’s Hospital, Vancouver, BC; 9 Department of Pediatrics, Kingston Health Sciences Centre, Kingston, ON; 10 Northern Ontario School of Medicine University, Thunder Bay, ON

**Keywords:** *Haemophilus influenzae*, invasive disease, bacteremia, meningitis, serotype a, serotype b, serotype f, non-typeable, children

## Abstract

**Background:**

There has been dramatic reduction in *Haemophilus influenzae* serotype b (Hib) since introduction of Hib vaccines, but children still experience serious invasive *Haemophilus influenzae* (Hi) disease caused by various serotype and non-typeable bacteria. The object of this study was to describe the serotype distribution and clinical spectrum of Hi bacteremia in children admitted to Canadian hospitals.

**Methods:**

All children with Hi bacteremia admitted 2013 through 2017 to 10 centres across Canada were included. Demographic, clinical, treatment and outcome data were collected.

**Results:**

*Haemophilus influenzae* bacteremia occurred in 118 children of median age 12 months (inter-quartile range: 7–48 months). Forty-three (36%) isolates were non-typeable (NTHi) and 8 were not typed. Of the 67 typeable (THi), Hia (*H. influenzae* serotype a) (n=36, 54%), Hif (serotype f) (n=19, 26%) and Hib (serotype b) (n=9, 13%) dominated. The THi was more likely than NTHi bacteremia to present as meningitis (*p*<0.001), particularly serotype a (*p*=0.04) and less likely to present as pneumonia (*p*<0.001). Complicated disease (defined as intensive care unit admission, need for surgery, long-term sequelae or death) occurred in 31 (26%) cases and were more likely to have meningitis (*p*<0.001) than were those with uncomplicated disease.

**Conclusion:**

In the era of efficacious conjugate Hib vaccines, NTHi, Hia and Hif have emerged as the leading causes of invasive Hi in Canadian children, with Hia being most likely to result in meningitis and complicated disease. A vaccine for all NTHi and THi would be ideal, but knowledge of the current disease burden from circulating strains will inform prioritization of vaccine targets.

## Introduction

*Haemophilus influenzae* serotype b (Hib) was overwhelmingly the leading cause of invasive Hi disease until the introduction of the Hib vaccines into the routine childhood immunization schedule in the United States (US) and Canada in the late 1980s ((1)). This was followed by the emergence of Hi serotype a, particularly in Indigenous children in Canada and in Alaska ((2–4)). Recent publications from the US reported an increase in the incidence of invasive Hi disease in children, possibly due to an increase in the incidence of cases due to non-typeable Hi (NTHi) ((5,6)). We sought to describe the serotype distribution and clinical spectrum of Hi bacteremia in Canadian children across several provinces and to determine factors associated with complicated disease.

## Methods

### Study population and design

Ten centres within the Pediatric Investigators Collaborative Network on Infections in Canada (PICNIC) retrospectively enrolled all hospitalized children younger than 18 years of age with blood culture isolates of Hi from January 1, 2013, through December 31, 2017. For nine centres (London, Ontario; Hamilton, Ontario; Ottawa, Ontario; Kingston, Ontario; Winnipeg, Manitoba; Edmonton, Alberta; Vancouver, British Columbia; Halifax, Nova Scotia and Montréal, Québec), this was a sub-study arising from a retrospective cohort of gram-negative bacteremia, while the tenth centre (Sioux Lookout, Ontario) was added for this sub-study due to their known high incidence of Hi infections. Ethics approval was obtained at each participating centre and the need for parental consent was waived.

### Study definitions

The focus of infection was classified as meningitis, pneumonia, epiglottitis, skin and soft tissue infection, osteoarticular infection, other or none (isolated bacteremia with no focus). Multifocal disease was defined as bacteremia with two or more foci.

Disease was defined as complicated if any of the following occurred related to Hi disease: intensive care unit (ICU) admission; organ failure; surgical interventions including amputations for purpura fulminans or drainage of purulent collections (arthrocentesis did not qualify unless performed more than once); complications relating to disease focus including motor deficits, seizures, hydrocephalus, visual or hearing deficits, or necrotizing skin or lung infections; and death.

### Serotyping

Serotyping of isolates was completed using monovalent antisera at reference laboratories. Strains were classified by capsular type (a to f) or as non-typeable. When serotyping was not available, the isolates were recorded as not typed.

### Data collection

Demographic, clinical, microbiological, treatment, outcome and follow-up data were extracted from medical records and entered into REDCap (Research Electronic Data Capture) tools hosted at the University of Alberta by each participating centre ((7)).

### Statistical analysis

Descriptive analysis was conducted. Chi-square or Fisher’s exact test was used to compare categorical variables and nonparametric tests were used to compare continuous variables. Univariate analysis was used to explore potential factors associated with Hia disease and complicated disease course. Variables with a univariate *p* value of ≤0.2 and potential confounding factors (e.g. age and sex) were considered for inclusion in multivariable logistic regression model aimed at determining independent risk factors for complicated disease. The IBM SPSS version 28 was used for statistical analysis.

## Results

There were 118 cases of Hi bacteremia of which 74 (63%) were male (**Table 1**). The median age was 12 months (interquartile range [IQR]: 7–48 months) with 7 cases (6%) being neonates and 25 (21%) being 5 years or older.

**Table 1:** Demographic, clinical, microbiological and outcome-related patterns across serotypes in paediatric population, Canada

Features^a^	Total N=118	THi (a–f) n=67	Hia n=36	Hib n=9	Hic n=1	Hie n=3	Hif n=18	NTHi n=43	Untyped n=8
**Demographics**									
Median age in months, [IQR]^b^	12, [7–48]	12, [7–36]	12, [7–24]	7, [6–12]	8	48, [12–108]	18, [12–60]	24, [5–60]	30, [7–96]
Age 1 month or younger	7 (6%)	2 (3%)	0 (0%)	1 (11%)	0 (0%)	0 (0%)	1 (6%)	4 (9%)	1 (13%)
Age younger than 12 months	43 (36%)	24 (36%)	14 (39%)	5 (56%)	1 (100%)	0 (0%)	4 (22%)	17 (40%)	2 (25%)
Age younger than 24 months	69 (58%)	45 (67%)	26 (72%)	8 (89%)	1 (100%)	1 (33%)	9 (50%)	20 (47%)	4 (50%)
Age younger than 60 months	93 (79%)	59 (88%)	32 (89%)	9 (100%)	1 (100%)	2 (67%)	13 (72%)	31 (72%)	5 (63%)
Male sex	74 (63%)	38 (57%)	16 (44%)	9 (100%)	1 (100%)	1 (33%)	11 (61%)	30 (70%)	6 (75%)
**Clinical foci^c^**									
Meningitis	25 (21%)	21 (31%)	14 (39%)	3 (33%)	1 (100%)	0 (0%)	3 (17%)	3 (7%)	1 (13%)
SSTI	8 (7%)	8 (12%)	4 (11%)	4 (44%)	0 (0%)	0 (0%)	0 (0%)	0 (0%)	0 (0%)
Osteoarticular infection	6 (5%)	6 (9%)	4 (11%)	2 (22%)	0 (0%)	0 (0%)	0 (0%)	0 (0%)	0 (0%)
Pneumonia	41 (35%)	13 (19%)	5 (14%)	0 (0%)	0 (0%)	1 (33%)	7 (39%)	23 (53%)	5 (63%)
Epiglottitis	1 (1%)	1 (1%)	0 (0%)	0 (0%)	0 (0%)	0 (0%)	1 (6%)	0 (0%)	0 (0%)
Infective endocarditis^d^	1 (1%)	0 (0%)	0 (0%)	0 (0%)	0 (0%)	0 (0%)	0 (0%)	1 (2%)	0 (0%)
Multifocal disease^c^	7 (6%)	7 (10%)	5 (14%) 1) CNS/IE 2) CNS/mastoiditis 3) CNS/pneumonia 4) Pneumonia/OI 5) SSTI-suppurative myositis/fasciitis	0 (0%)	0 (0%)	0 (0%)	2 (11%) 1) Men/IE 2) Men/OI	0 (0%)	0 (0%)
Isolated bacteremia	29 (25%)	11 (16%)	4 (11%)	0 (0%)	0 (0%)	2 (67%)	5 (28%)	16 (37%)	2 (25%)
**Microbiology**									
Number of isolates with laboratory confirmed viral co-infection^e^	32 (27%)	14 (21%)	10 (28%)	1 (11%)	0 (0%)	0 (0%)	3 (17%)	15 (35%)	1 (13%)
Number of isolates with ampicillin resistance	25/106 (24%)	6/63 (10%)	0/34 (0%)	2/8 (25%)	0/1 (0%)	0/3 (0%)	4/17 (24%)	18/35 (51%)	1/8 (13%)
**Outcome**									
Median antibiotic duration^a^ (days), [IQR]	13.5, [10–23]	14, [10–28]	15, [11–29]	21, [10–31]	53	11, [11–11]	13, [9–16]	13, [10–21]	10, [10–19]
ICU admission^f^	39 (33%)	21 (31%)	13 (36%)	1 (13%)	1 (100%)	0 (0%)	6 (33%)	14 (33%)	4 (50%)
Complicated course^f^ [% with CNS complication]	31 (26%) [18/31 (58%)]	24 (36%) [15/24 (63%)]	14 (39%) [11/14 (79%)]	2 (22%) [1/2 (50%)]	1 (100%) [1/1 (100%)]	0 (0%) [0 (0%)]	7 (39%) [2/7 (29%)]	6 (14%) [2/6 (33%)]	1 (13%) [1/1 (100%)]
Median length of stay^g^ (days), [IQR]	10, [5–21]	9, [4–16.5]	10, [4–18]	13, [7–20]	55	1, [0–5]	7, [2–13]	12, [7–30]	12, [8–30]
Death	2 (2%)	2 (3%)	2 (6%)	0 (0%)	0 (0%)	0 (0%)	0 (0%)	0 (0%)	0 (0%)
Death/sequelae^h^	17 (14%)	14 (21%)	9 (25%)	2 (22%)	1 (100%)	0 (0%)	2 (11%)	2 (5%)	1 (13%)

Abbreviations: CNS, central nervous system; Hia, *Haemophilus influenzae* serotype a; Hib, *Haemophilus influenzae* serotype b; Hic, *Haemophilus influenzae* serotype c; Hie, *Haemophilus influenzae* serotype e; Hif, *Haemophilus influenzae* serotype f; ICU, intensive care unit; IE, infective endocarditis; IQR, interquartile range; NTHi, non-typeable *Haemophilus influenzae*; OI, osteoarticular infections; SSTI, skin and soft tissue infection; THi, typeable *Haemophilus influenzae*

^a^ Values shown as totals (%) for categorical variables and medians with IQRs for continuous variables

^b^ Pairwise comparison of medians across serotype distribution was not significantly different after adjusting by Bonferroni correction for multiple tests

^c^ Unless listed as multifocal disease (more than two systems) or same system disease in distant sites, the focus listed was the sole focus identified during the admission. A sinusitis case was classified as pneumonia given that both are respiratory foci. Skin and soft tissue infections included unspecified cellulitis, facial cellulitis (n=3) and cellulitis with fasciitis (n=1; caused by Hia). The latter case with associated suppurative myositis

^d^ Infective endocarditis refers to endovascular infection (n=1) or endocarditis (n=2); the latter occurred in absence of preceding cardiac disease. Two cases caused by Hia and Hif respectively were multifocal with CNS involvement. The endovascular infection occurred in an adolescent (without immunodeficiency) who presented with Lemierre syndrome with thrombosis of both internal jugular veins with associated Hif meningitis (patient also grew *Streptococcus anginosus* in blood)

^e^ Thirty-nine (33%) of patients had preceding viral symptoms. Thirty-two had viral polymerase chain reaction (PCR) test confirmation as enterovirus/rhinovirus (n=12), influenza (n=8), respiratory virus (n=5), parainfluenza (n=3), human coronavirus (n=1) and human metapneumovirus (n=1). Two children had multiple viral infections including 1) enterovirus/rhinovirus and adenovirus and 2) enterovirus/rhinovirus and bocavirus

^f^ Courses were considered complicated if there was ICU requirement or if disease-specific complications (e.g. ventriculitis, necrotizing pneumonia, need for infectious foci-related surgery, longer than usual course of antimicrobial therapy for a given focus. Complications in THi were mainly related to CNS infections. For NTHi, two of six children with complicated courses had CNS complications (ventriculitis and seizures). Other complications in NTHi were due to pneumonia (three needing chest tube, one of whom was ventilated) and another developing shock requiring ICU admission. Complications arising from treatment not included in disease complications included: *Clostridium difficile* colitis in an oncology patient and a central line infection. Among THi, there was one treatment-related complication (cholestasis from ceftriaxone that was not included in complicated disease criteria)

^g^ Mean length of stay for ICU admissions was three days [IQR: 2–10]

^h^ Sequelae includes all neurological complications arising from Hi disease persisting at discharge or end of antibiotic therapy (including those who received outpatient parenteral antibiotics). Central nervous system complications (i.e. ventriculitis, empyema, cerebritis) that resolved prior to the end of therapy were not included as sequelae

**Figure 1:**
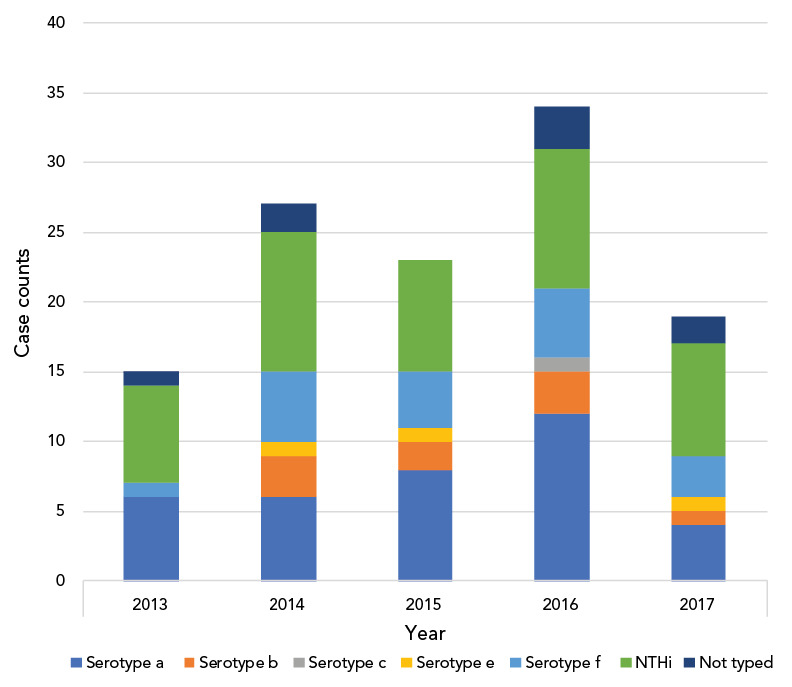
*Haemophilus influenzae* serotype distribution by year in paediatric population, Canada Abbreviation: NTHi, non-typeable *Haemophilus influenzae*

**Figure 2:**
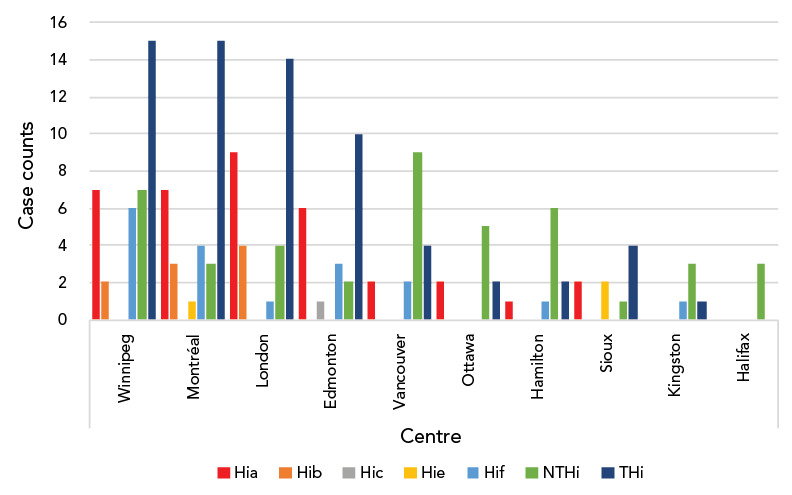
Serotype-specific distribution of *Haemophilus influenzae* invasive disease in ten centres across Canada^a^ Abbreviations: NTHi, non-typeable *Haemophilus influenzae*; THi, typeable *Haemophilus influenzae* disease including disease caused by serotypes a, b, c, e, f ^a^ Centre names: Winnipeg Children’s Hospital (Winnipeg, Manitoba); Montreal Children’s Hospital (Montréal, Québec); Children’s Hospital of Western Ontario (London, Ontario); Stollery Children’s Hospital (Edmonton, Alberta); British Columbia Children’s Hospital (Vancouver, British Columbia); Children’s Hospital of Eastern Ontario (Ottawa, Ontario); McMaster Children’s Hospital (Hamilton, Ontario); Sioux Lookout (Sioux Lookout, Ontario); Kingston Health Sciences Centre (Kingston, Ontario); IWK Health Centre (Halifax, Nova Scotia)

Typing was available for 110 Hi isolates, of which 67 (61%) were typeable *Haemophilus influenzae* (THi) and 43 (39%) were NTHi. Serotypes a, f and b were the leading serotypes accounting for 36 (54%), 18 (27%) and 9 (13%) of THi respectively (Table 1). Age distribution across serotypes was not significantly different (Table 1). Case numbers were similar across the years of the study (mean: 23.6±7.33), with a peak in 2016 (**Figure 1**). Centre-specific contributions are shown in **Figure 2**. Centres in Winnipeg, Montréal and London contributed most Hi cases, with disease predominantly caused by THi.

Yearly case frequency ranged from 15–34 cases annually with the peak frequency occurring in 2016. Non-typeable *H. influenzae* followed by serotype a then serotype f disease were the leading causes of Hi disease in 2013, 2014 and 2017. Serotype a followed by NTHi and then serotype f were the leading causes in 2015 and 2016.

Winnipeg, Montréal, London and Edmonton had the most total cases of THi. Serotypes a and f accounted for most cases of THi across all centres, with the exception of London, which identified predominantly cases of serotypes a and b. Frequency of both Hia and Hib were highest in London. Vancouver, Winnipeg, Ottawa and Hamilton had the most cases of NTHi.

Thirty-one (27%) of 116 children with available clinical details (data missing for two cases) had at least one underlying medical condition which included prematurity (n=11; 9%), malignancy (n=11; 9%), immunodeficiency (n=9; 8%) and genetic or metabolic syndrome (n=8; 7%). Of the 108 children with serotyping who had available data, 13/67 (19%) of THi versus 15/41 (35%) with NTHi had an underlying condition (*p*=0.048). Among six Hib cases with available information on vaccine history, five (83%) had not received any Hib vaccine (n=3) or had inadequate number of doses for age (n=2).

Twenty-five (24%) of 106 isolates with susceptibility reporting available were resistant to ampicillin while none were resistant to ceftriaxone (Table 1). The NTHi isolates were more likely to demonstrate resistance to ampicillin than were THi (n=18/35, 51% vs. n=6/63, 9%; *p*<0.001).

Among 67 children with THi, 11 (16%) had isolated bacteremia with no focus. Forty-nine had a single focus, including meningitis (n=21), pneumonia (n=13), skin and soft tissue infection (n=8), osteoarticular infection (n=6) and epiglottitis (n=1) (Table 1). Multifocal disease occurred in seven other children (five due to Hia and two due to Hif), with five cases having meningitis as one of the foci.

Among the 43 children with NTHi, 16 (37%) had isolated bacteremia with no focus when compared with 11/67 (16%) of THi (*p*=0.013). The remaining 27 children had a single focus including pneumonia (n=23), meningitis (n=3) and infective endocarditis (n=1) (Table 1).

Meningitis was more common with THi than NTHi (n=26/67, 39% vs. n=3/43, 7%; *p*<0.001) and was equally common with Hia and Hib (47% vs. 33%; *p*>0.05). Pneumonia (as a single focus) was more common with NTHi than THi (n=23/43, 53% vs. n=13/67, 19%; *p*<0.001).

The median duration of antibiotic therapy in the entire cohort was 13 days [IQR: 10–23] with prolonged duration for osteoarticular infection and meningitis with median duration of 30 days [IQR: 26–41] and 24.5 days [IQR: 12–43], respectively (Table 1). Thirty (26%) of 113 cases with available information who received a median of 14 days (IQR: 11–28) in total were transitioned from parenteral after a median of 7 days (IQR: 4–13) to oral antibiotics to complete remainder as oral therapy.

Complicated disease was more common with THi than with NTHi (n=24/67, 36% vs. n=6/43, 14%; *p*=0.015). The fatal cases were both caused by Hia and occurred in an infant with meningitis and endocarditis despite no congenital heart disease and in a four-year-old with meningitis complicated by subdural empyema. One infant with Hib meningitis required bilateral limb amputations due to purpura fulminans but survived. The composite outcome of mortality or sequelae at discharge was significantly associated with Hia as compared to non-Hia disease (n=9/36, 25% vs. n=7/74, 9%; *p*<0.001) (see **Appendix**).

In the univariate analysis (after adjusting for multiple comparisons), Hi cases with meningitis (*p*<0.001) were more likely to have a complicated clinical course whereas those with isolated bacteremia were less likely (*p*<0.001) (**Table 2**). In the multivariate analysis, meningitis (*p*<0.001) predicted a complicated disease course after controlling for age (*p*>0.05) and Hia serotype (*p*>0.05) (Table 2).

**Table 2:** Univariate and multivariate analysis of factors associated with complicated *Haemophilus influenzae* disease

Features^a^	All N=118	Complicated disease n=31	Uncomplicated disease n=87	Significance *p*<0.006^b^	Multivariate analysis^c^	
					Odds ratio, [95% CI]	*p* value
**Demographics**						
Males	74 (63%)	21 (68%)	55 (63%)	0.800	1.27, [0.445–3.62]	0.67
Median age (months), [IQR]	12, [7–48]	12, [6–36]	12, [9–60]	0.021	1.02, [1.00–1.03]	0.12
Patients younger than 1 year	43 (36%)	15 (48%)	28 (32%)	0.107	N/A	N/A
**Clinical spectrum**						
Isolated bacteremia	29 (25%)	1 (3%)	30 (34%)	<0.001	14.77, [5.22–41.76]	<0.001
Meningitis	29 (25%)	20 (65%)	9 (10%)	<0.001	N/A	N/A
Pneumonia	43 (36%)	11 (35%)	33 (38%)	0.809	N/A	N/A
**Microbiology**						
NTHi	43 (36%)	6 (19%)	25 (29%)	0.029	N/A	N/A
Hia	36 (31%)	14 (45%)	21 (24%)	0.028	N/A	N/A
**Inflammatory markers**						
Median peak white cell count, [IQR]	18.1, [12.1–26.3]	21.3, [16.3–30.3]	16.7, [11.8–21.3]	0.072	N/A	N/A
Median C-reactive protein (mg/L), [IQR]	155, [72–223]	194, [134–301]	114, [65–194]	0.03	N/A	N/A
**Treatment**						
Median duration of antibiotic therapy (days), [IQR]	14, [10–23]	26, [11–44]	13, [10–20]	0.039	N/A	N/A
Median length of stay (days), [IQR]	10, [5–21]	18.5, [9–43.5]	9, [4–16]	0.043	N/A	N/A

Abbreviations: CI, confidence interval; Hia, *Haemophilus influenzae* serotype a; IQR, interquartile range; N/A, not applicable; NTHi, non-typeable *Haemophilus influenzae*

^a^ Values shown as totals (%) for categorical variables and medians with interquartile ranges (IQR) for continuous variables. Chi-square or Fischer exact test for categorical variables; Kruskal-Wallis test for continuous variables

^b^ Significance level was adjusted for multiple comparisons using Bonferroni correction

^c^ In the multivariate analysis, after controlling for age and Hia status, meningitis remained an independent predictor of a complicated course (*p*<0.001)

## Discussion

This multicentre study from 10 sites across Canada provides insight into the current serotype distribution of Hi bacteremia in Canadian children. Prior to the era of Hib vaccine, invasive Hi disease was very rare in children over four years of age, while approximately one quarter of our cases were older. Invasive and non-invasive Hi disease from 2013 to 2019 has been reviewed from epidemiological data from a single Canadian site with a predominant Indigenous population (Sioux Lookout) ((8)). At this centre, invasive Hi disease was identified in 10 children under 4 years, in two aged 5–15 years and in eight aged 16 years and older ((8)), suggesting a bimodal distribution which differs from the pre-Hib vaccine era where younger children were primarily affected. Our data highlight the pediatric centres in Winnipeg, Montréal, London and Edmonton as the highest contributors to THi, and may reflect that these centres are referral centres for large communities of Indigenous children as well as communities with reduced Hib vaccine uptake. The Hib cases still accounted for almost 10% of cases in the current study. As expected, Hib occurred predominantly in unimmunized or under-immunized children.

Serotypes a and f were the most common, accounting for 55% and 25% of THi, respectively, consistent with recent literature ((4,6)). While Hia meningitis was significantly associated with a complicated course, it otherwise mirrored the Hib experience in terms of age and clinical syndromes. Children with Hif were often older with pneumonia as the focus of infection.

The less virulent NTHi accounted for just over one third of all Hi bacteremic events, where fortunately the incidence of complicated disease was only 4%. The NTHi cases presented as isolated bacteremia or pneumonia and rarely as central nervous system disease, in keeping with recent US data ((5,6)). Comorbid medical conditions were a risk factor for developing NTHi disease. In a retrospective surveillance study of NTHi invasive disease in children and adults in the Netherlands, comorbid conditions in children, including immunocompromise, malignancy, neurological disease and other conditions, were identified in a large proportion of NTHi cases (n=327/396, 83%) over a seven-year span ((9)).

It is exciting that Phase I trials of a Hia vaccine will begin in Canada in 2023 ((10)) (*personal communication, M. Ulanova, Canadian Immunization Research Network, 2022*). A vaccine has been shown to be cost-effective in the Canadian territory of Nunavut, given the high incidence of Hia among Indigenous children and their high risk of disease ((11)). Post-marketing studies of the multivalent pneumococcal vaccines that employ protein D from NTHi as the carrier protein suggest that they prevent some otitis media due to NHTi diseases, so it is plausible that these vaccines may also prevent Hi bacteremia ((12)).

## Limitations

A significant limitation of our study is that it did not capture ethnicity/race data as this variable is not reliably recorded in health records in Canada. This is especially important given the established high rates of Hia in Indigenous populations ((4)). Our study may underestimate the burden and spectrum of Hi disease given that it was limited to bacteremic cases; blood cultures are not always drawn prior to administration of antibiotics and can be falsely negative if an inadequate volume is obtained. As neurodevelopmental and long-term follow-up data were not uniformly available, we reported sequelae documented at or before discharge.

## Conclusion

Our study provides detailed clinical comparisons of THi and NTHi, highlighting serotype-specific clinical patterns and outcomes. Although there has been dramatic reduction in Hib since introduction of Hib vaccines, children still experience serious invasive Hi disease caused by both NHTi and by non-b serotypes, especially Hia. Preventive strategies are needed to reduce the morbidity associated with this disease.

## Appendix

**Table A1:** Univariate analysis of factors associated with *Haemophilus influenzae* serotype a invasive disease

Features^a^	All N=110 (excluding untyped)	Hia n=36	Non-Hia n=74	Univariate analysis significance *p* value<0.006^b^
**Demographics**				
Females	42 (38%)	20 (56%)	22 (30%)	0.009
Median age (months), [IQR]	12, [7–48]	12, [7–24]	12, [7–60]	0.155
**Clinical spectrum**				
Meningitis	29 (26%)	17 (47%)	12 (16%)	<0.001
Pneumonia	38 (35%)	7 (19%)	31 (42%)	0.021
Bacteremia	27 (25%)	4 (11%)	23 (31%)	0.032
Complicated disease	30 (27%)	14 (39%)	16 (22%)	0.002
**Treatment/outcome**				
Median duration of antibiotic therapy (days), [IQR]	14, [10–24]	15, [11–29]	13, [10–21]	0.097
Median length of stay (days), [IQR]	14, [10–24]	10, [4–18]	10.5, [5–21]	0.987
Death/sequelae^c^	16 (15%)	9 (25%)	7 (9%)	<0.001

Abbreviations: Hia, *Haemophilus influenzae* serotype a; IQR, interquartile range

^a^ Values shown as totals (%) for categorical variables and medians with interquartile ranges (IQR) for continuous variables. Chi-square or Fisher exact test for categorical variables; Kruskal-Wallis test for continuous variables

^b^ Significance level was adjusted for multiple comparisons using Bonferroni correction

^c^ Sequelae includes all neurological complications arising from *Haemophilus influenza* disease persisting at discharge or end of antibiotic therapy (including those who received outpatient parenteral antibiotics). Central nervous system complications (i.e. ventriculitis, empyema, cerebritis) that resolved prior to the end of therapy were not included as sequelae
